# In Vitro Evaluation of the Mechanical Properties of Posterior Adhesive Restorations Fabricated Using Three Different Techniques

**DOI:** 10.3390/polym17101340

**Published:** 2025-05-14

**Authors:** Cem Peskersoy, Gozde Acar

**Affiliations:** Department of Restorative Dentistry, Faculty of Dentistry, Ege University, 35040 Izmir, Türkiye; gozde.acar@ege.edu.tr

**Keywords:** three-dimensional dental printing, adhesive bridge, color stability, fiber mesh, finite element stress analysis, indirect restoration

## Abstract

This study evaluates the optical properties and mechanical durability of adhesive restorations fabricated using different techniques for the treatment of single-tooth loss in the posterior region after an aging process. Sixty extracted human teeth (thirty molars and thirty premolars) were restored using three different fabrication methods: 3D-printed resin restorations, fiber mesh-reinforced direct composite restorations, and indirect composite restorations. Color stability was assessed using a spectrophotometer, and fracture resistance was measured using a universal testing machine. Finite element stress analysis (FEA) was conducted to validate mechanical test results under simulated intraoral conditions. The fiber-reinforced composite group exhibited the highest fracture resistance (1057.91 MPa), while 3D-printed restorations showed the lowest (*p* < 0.05). Regarding color stability, the fiber-reinforced group demonstrated the highest ΔE^00^ values (ΔE^00^ = 1.71), differing significantly from the other groups, while the 3D-printed and indirect composite restorations showed no significant difference. Mechanical test results were consistent with FEA findings. These results indicate that fiber reinforcement enhances mechanical durability in high-load-bearing areas, while 3D-printed restorations may not yet be suitable for posterior regions. However, their potential use in anterior restorations, where occlusal forces are lower, warrants further investigation to improve material properties.

## 1. Introduction

Congenitally missing teeth represent one of the most frequently encountered developmental anomalies in contemporary dental practice. The absence of teeth can be attributed to congenital factors, trauma, or extractions. Excluding third molars, the most commonly missing teeth are the maxillary lateral incisors and mandibular second premolars [1]. These missing teeth can cause significant functional and esthetic concerns, necessitating a multidisciplinary treatment approach [1].

Various treatment modalities have been developed to rehabilitate single-tooth agenesis, including conventional fixed prostheses, implant-supported restorations, removable prostheses, and adhesive bridges [2]. The selection of the most appropriate treatment depends on several factors, including the patient’s overall health, financial considerations, and the dental and periodontal status of the adjacent teeth. Recently, with the increasing emphasis on minimally invasive approaches, adhesive bridge restorations have gained popularity as a preferred treatment option for single-tooth replacements [3].

Advancements in three-dimensional (3D) printing technology have introduced groundbreaking innovations in restorative dentistry, and 3D-printed adhesive bridges offer advantages over conventional techniques, such as rapid production, cost-effectiveness, patient-specific customization, and digital data storage. Furthermore, this technology enables improved marginal and occlusal adaptation, reduces errors in the fabrication process, and enhances esthetic outcomes [4]. However, the mechanical strength, long-term clinical success, and prognosis of 3D-printed adhesive bridges under occlusal loading remain inadequately documented in the literature. Recent reviews, however, have emphasized ongoing advancements in resin formulations and 3D printing technologies aimed at improving the mechanical performance of dental restorations [5,6]. Therefore, comprehensive in vitro studies are required to evaluate the effectiveness of this emerging technique, assess the optical properties of the materials used, and determine their durability under intraoral dynamic conditions.

The present study aims to compare the mechanical strength and optical properties of adhesive bridges fabricated using different methods in posterior single-tooth replacement cases. This study will evaluate the fracture resistance of restorations produced using three distinct fabrication techniques and validate the findings through finite element stress analysis (FEA). The results of this research are expected to contribute to the development of more durable and long-lasting adhesive bridge designs in restorative dentistry.

Three main hypotheses were proposed:

1. Adhesive restorations fabricated using three different methods will exhibit similar fracture resistance in compression tests, with no statistically significant differences among the groups.
2. After the aging process, the color changes of the adhesive restorations produced using the three different methods will remain within clinically acceptable limits, with no statistically significant differences between the groups.
3. The static and dynamic compression resistance values calculated using FEA will be consistent with the results obtained from mechanical tests.

## 2. Materials and Methods

### 2.1. Preparation and Grouping of Samples

Prior to the study, ethical committee approval was obtained from the Ege University Medical Research Ethics Committee with the approval number 23-12.1T/66 dated 28 December 2023, for the extracted human teeth to be used in the study.

For the study, 60 sound molar and premolar teeth, extracted for periodontal or orthodontic reasons, were selected from the Department of Oral and Maxillofacial Surgery. Teeth with visible fractures or caries were excluded. The selected teeth were stored in distilled water until cleaning, after which soft and hard tissue remnants were removed using a scalpel. Subsequently, the teeth were immersed in a fresh distilled water solution.

Standard-sized, reusable rectangular blocks were fabricated using a 3D printer with flexible TPU filament (Porima, Yalova, Türkiye) to accommodate the teeth. Key–lock models of the same material were created to ensure standardized tooth spacing. The teeth were grouped into pairs, consisting of one premolar and one molar, to serve as abutments for the adhesive bridges, ensuring similarity in crown size and form.

The 30 pairs were randomly assigned into three groups (n = 10) according to the fabrication method: 3D printing (3D), fiber-reinforced direct composite (FC), and indirect composite (IC). The roots of the grouped teeth were covered with transparent tape at the cemento-enamel junction to keep the crowns exposed. The teeth were then embedded in acrylic resin (Duracryl Self Cure, Duradent Erk Dental, İzmir, Türkiye) at the cemento-enamel junction level. Once polymerization was complete, the teeth were removed from the blocks, and the transparent tape was removed. A silicone impression material (Zhermack, Badia Polesine, Italy) was added to the sockets before reinserting the teeth to simulate the periodontal ligament effect.

Modified two-surface inlay cavities were prepared on the proximal surfaces of the teeth facing the edentulous space using a high-speed handpiece (KaVo S608C Smart, KaVo Dental GmbH, Biberach, Germany) under water cooling. These cavities had a box-like configuration without a proximal step. The cavity dimensions for molar teeth were standardized as 3 mm in bucco-lingual width, 3 mm in occluso-cervical depth, and 7 mm in mesio-distal length. For premolar teeth, the dimensions were set at 3 mm in bucco-lingual width, 3 mm in occluso-cervical depth, and 4 mm in mesio-distal length.

### 2.2. Preparation of Restorations

In this study, different restorative methods used for the treatment of single-tooth deficiencies were compared, including direct, indirect, and semi-direct techniques. The planned treatment approaches included fabrication using a dental 3D printer (indirect method), woven fiber-reinforced resin composite application (direct method), and laboratory composite restoration on teeth (semi-direct method).

#### 2.2.1. 3D Group

In the group involving 3D-printed restorations, digital impressions of the samples were obtained using an intraoral scanner (TRIOS^®^ 3, 3Shape, Copenhagen, Denmark) and saved in STL format.

The restoration designs were created using DWOS software (DentalWings software v. 2023.2.1, Montreal, QC, Canada). Sample numbers were assigned as patient identifiers, and the corresponding teeth and restoration types were selected. The cavity margins for premolar and molar teeth were defined, and the pontic and connector regions were designed accordingly.

Following the completion of the digital design, the STL files were transferred to slicing software compatible with the 3D printer (Bego CAM Creator v5.1.11, Bremen, Germany). Support and connection points were designated before transferring the files via USB to the Bego Varseo XS (Bego, Bremen, Germany) 3D printer.

Ten samples in this group were fabricated using the Digital Light Processing (DLP) technique at a 405 nm wavelength, with a resolution of 50 µm and a printing speed of 30 mm/h. Varseo Smile Crown Plus (Bego, Bremen, Germany) permanent restoration resin was used for fabrication. Prior to the printing process, the Bego Varseo XS 3D printer was calibrated following the manufacturer’s specifications. The calibration procedure involved several steps to ensure optimal printing accuracy and material performance. First, platform leveling was performed using the integrated alignment tool, and deviations were verified to remain within a ±10 µm tolerance range. Subsequently, the resin vat was inspected and cleaned to prevent any particulate contamination that could affect the print quality. The exposure parameters were then adjusted according to the optical properties of the VarseoSmile Crown Plus resin, setting the exposure time at 8.5 s per layer for standard 50 µm layers and 15 s for initial layers. Dimensional accuracy was verified by printing a standard calibration model provided by the manufacturer, and measurements confirmed a tolerance within ±5% of the intended dimensions. Finally, the light intensity at the build platform surface was measured using a radiometer, ensuring that the output matched the specified 5.2 mW/cm^2^ at 405 nm wavelength. These calibration procedures were crucial for ensuring the reproducibility and reliability of the printed restorations in this study. Once the printing process was completed, the restorations were carefully removed from the building platform.

The printed restorations were placed in a compartmentalized plastic container filled with 96% ethanol solution and cleaned for 480 s in an ultrasonic bath (Foshan Adelson Medical Devices Co., Foshan, China). The specimens were then carefully dried to observe with light microscopy (DM 500, Leica, Wetzlar, Germany) to detect any micro-gaps or production faults which may cause wrong measurements. The 3D-printed restorations with micro-gaps caused by shrinkage of the 3D resin or insufficient curing which were detected during visual inspection or during storage in ethanol were re-manufactured without cementation. For post-curing, the restorations were placed in the Bego Otoflash (Bego, Bremen, Germany) unit. Under a nitrogen gas atmosphere (1.0–1.2 bar), the restorations were subjected to 1500 flashes at a frequency of 10 Hz. The restorations were then flipped, and an additional 1500 flashes were applied to the opposite surface.

#### 2.2.2. Indirect Composite Group

For the group restored using the indirect technique, molar and premolar teeth embedded in acrylic were completely covered with Teflon tape. Composite resin (Signum, Heraeus Kulzer, Hanau, Germany) was applied in layers of 1.5–2 mm using a freehand technique to achieve natural morphology, with each layer polymerized using a light-curing device (Woodpecker Led C, Foshan Ltd., Zhengzhou, China). Following polymerization, the restorations underwent post-curing in the Bego Otoflash (Bego, Bremen, Germany) unit under a nitrogen gas atmosphere at 10 Hz, with 1500 flashes applied twice.

For surface preparation, the enamel margins of the cavity were etched with 37% orthophosphoric acid (Heraeus Kulzer, Hanau, Germany) for 30 s, followed by rinsing with pressurized water and gentle drying with an air spray. Next, 4% hydrofluoric acid (HF) (Heraeus Kulzer, Hanau, Germany) was applied to the inner surfaces of the restorations in contact with the cavity. The acid was rinsed off with pressurized water and gently dried with an air spray. Finally, silane (Gluma Ceramic Primer, Heraeus Kulzer, Hanau, Germany) was applied to the same surfaces, left for 20 s, and then gently air-dried to prepare the restoration for adhesive bonding. After completing the preparation of the cavity and restoration surfaces before cementation, G-Cem One self-adhesive resin cement (GC, Tokyo, Japan) was applied to the inner surface of the restoration. The restoration was adapted to the tooth using finger pressure. After removing excess cement, all surfaces were polymerized for 40 s using the same light-curing device.

For the removal of residual cement after polymerization, polishing discs (Tor, Moscow, Russia) and rubber polishers were used. To enhance wear resistance and achieve a uniform surface smoothness across all restorations, Optiglaze Color (GC, Tokyo, Japan) was applied with a brush and polymerized for 20 s on each surface.

#### 2.2.3. Woven Fiber and Direct Composite Group

In this study, for the group to be restored using the direct technique, selective enamel etching was performed using 37% orthophosphoric acid for 30 s, followed by rinsing with pressurized water and gentle air drying. As the bonding agent, Gluma Universal Bond (Heraeus Kulzer, Hanau, Germany) was actively rubbed into the cavity using a bond brush for 20 s, then gently air-dried and light-cured for 10 s.

To enhance the adaptation of the woven fiber within the cavities, a thin layer of flowable composite was applied, and an appropriately sized woven fiber was placed into the cavities, followed by polymerization of all surfaces for 40 s. Subsequently, another thin layer of flowable composite was applied and polymerized, and restorations were completed using a direct composite material. In the final step, to increase wear resistance and achieve a smooth surface finish, Optiglaze Color was applied and polymerized for 20 s on each surface.

### 2.3. Color Measurement

The initial color values of the samples were measured and recorded using the VITA Easyshade V (Vita Zahnfabrik, Bad Säckingen, Germany) spectrophotometer, which employs the CIElab color system. Subsequently, the samples were subjected to a total of 5000 thermal cycles between 5 °C and 55 °C, simulating approximately six months of intraoral aging. Each temperature change included a 10 s dwell time, and the transition time between temperature baths was set to 10 s.

Following the completion of the thermal cycling process, color measurements were repeated, and the color differences between the initial and final measurements were calculated using the CIEDE2000 (ΔE^00^) formula. In this study, the CIEDE2000 (ΔE^00^) formula was used to calculate color differences, as it is currently considered the most accurate and clinically relevant method for evaluating perceptible and acceptable color changes in dentistry. Compared to the traditional CIELAB (ΔE*ab) formula, CIEDE2000 provides better correlation with human visual perception by incorporating corrections for lightness, chroma, and hue non-uniformities. Additionally, CIEDE2000 introduces parametric weighting factors that adjust for viewing conditions, thereby offering a more refined and sensitive analysis of color discrepancies. These advantages made it the most appropriate choice for the color stability assessment in this study. Before each measurement, the device was calibrated according to the manufacturer’s instructions, and the measurements were taken from the buccal surface of the premolar tooth on the sample.

### 2.4. Application of Fracture Strength Test

**Figure 1:**
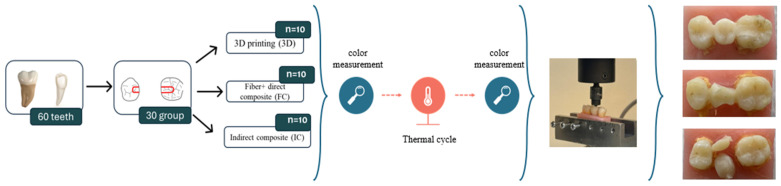
Scheme of the preparation procedure of samples.

The fracture resistance test was performed using a universal testing machine (Shimadzu AG-X, Kyoto Japan). During the test, a steel ball-shaped loading tip with a diameter of 10 mm, selected to fit the central fossa of the teeth, was positioned 1 mm above the central fossa of the pontic premolar in the restoration, and a maximum load of 2000 N/cm^2^ was applied. At the end of the test, the maximum load values (N) for all samples and the maximum stress values (MPa) recorded at the fracture point were documented, and the samples were photographed (Figure 1).

### 2.5. Finite Element Stress Analysis (FEA)

In this study, finite element stress analysis (FEA) was performed to validate the obtained fracture resistance results and assess their compatibility with clinical conditions. This analysis identified the contact points in restorations and adjacent teeth during mastication, simulating stress accumulation and displacement in force-applied regions. Thus, the accuracy and clinical relevance of the mechanical test results were evaluated. The mechanical properties of dental tissues and restorative materials used in FEA models were obtained from the literature, and each layer was individually modeled and transferred to the simulation environment (Table 1)

**Figure 2:**
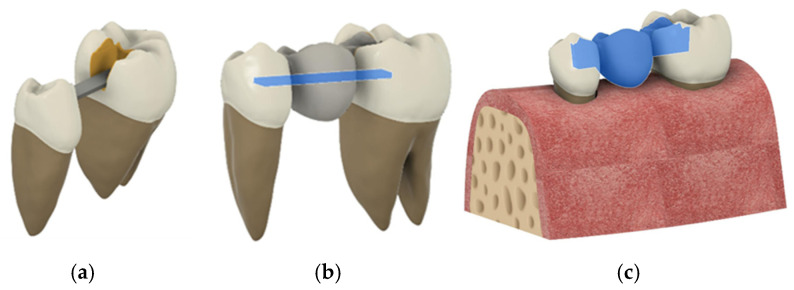
Transfer of used materials and biological tissues to the simulation area.

Two teeth (a mandibular first molar and a mandibular first premolar) that were allocated for the compressive strength test but not used in this study were digitized using high-resolution cone-beam computed tomography (CBCT). The obtained images were converted into solid models and meshed using a triangular element system. To enhance computational accuracy, an h-adaptive mesh convergence analysis was performed, and the optimal number of elements was determined. During the modeling process, virtual cavities were created for all three restoration types, ensuring that they included enamel and dentin tissues while excluding pulpal and periodontal areas. A suitable pontic model was selected and placed in the space of the missing tooth, and restorations in the adjacent cavities were designed according to the natural anatomy of the teeth (Figure 2).

Boundary conditions were defined based on loading scenarios that best represented natural masticatory forces. Contact points formed with maxillary molars were identified, and occlusal contact points in all three teeth were determined for static fracture testing at the central ridge of the pontic premolar and for dynamic analyses. A biting force of 300 N was applied parallel to the long axis of the models, and the total displacement values, maximum von Mises equivalent stress values, and stress distribution patterns were recorded. In the calculation of fracture resistance, linear fracture mechanics formulas were used to determine the estimated fracture resistance for each model. Consequently, differences in mechanical durability and stress distribution among various restoration groups were comprehensively analyzed, providing a detailed evaluation of fracture mechanisms.

### 2.6. Statistical Analysis

The fracture resistance, von Mises stress values, and color change data obtained from this study were analyzed using SPSS 27 statistical software (v. 27.1.0, IBM Corp, Chicago, IL, USA). The normality distribution of the data was assessed using the Kolmogorov–Smirnov test (*p* = 0.080), and the significance level was set at α = 0.05.

The data from the compressive strength test were analyzed using one-way analysis of variance (ANOVA) and post hoc Tukey’s HSD test. Differences in color measurements were evaluated using the Kruskal–Wallis non-parametric test. The correlation between the results of the finite element stress analysis (FEA) and the fracture values obtained from the ex vivo compressive strength test was examined using the Spearman correlation test.

## 3. Results

### 3.1. Evaluation of the Fracture Resistance (K_IC_) of the Samples

The normality of the fracture resistance (K_IC_) data obtained in the study was assessed using the Kolmogorov–Smirnov test. The test results indicated a significant value of *p* = 0.082, and since *p* > 0.05, the data were determined to follow a normal distribution.

Analysis of the obtained data showed that the highest fracture resistance was observed in the woven composite (FC) group, with a mean value of 1750.75 N, while the lowest fracture resistance was recorded in the 3D-printed restoration (3D) group, with a mean value of 206.45 N. The statistical relationship between the mean fracture resistance values of the three groups was analyzed using one-way ANOVA, revealing a significant difference among the groups (*p* < 0.05) (Table 2). To determine which groups contributed to this difference, a post hoc Tukey multiple comparison test was performed.

The Tukey test results indicated a statistically significant difference between the 3D and FC groups (*p* = 0.001). However, the differences between the 3D and indirect composite (IC) groups and between the FC and IC groups were not statistically significant (*p* = 0.064 and *p* = 0.158, respectively). The ranking of fracture resistance among the groups was determined as 3D < IC < FC, with a significant difference only between the 3D and FC groups. These findings suggest that woven fiber and direct composite restorations exhibit higher fracture resistance compared to the other groups.

### 3.2. Evaluation of Color Change Differences (ΔE^00^) in Samples

Color measurements of the samples were performed using a spectrophotometer before and after thermal cycling, and color change differences (ΔE^00^) were calculated using the CIEDE2000 formula. The analysis revealed that woven fiber and direct composite restorations (FC) exhibited the highest color change, whereas 3D-printed restorations (3D) demonstrated the lowest (Table 3). A statistically significant difference was found between the 3D and FC groups, while no significant difference was observed between the 3D and indirect composite (IC) groups.

Although various methods are available for calculating color differences in dentistry, the CIEDE2000 (ΔE^00^) formula is considered more reliable. The perceptibility and clinical acceptability thresholds are set at 0.8 and 1.8, respectively. Based on these thresholds, only the 3D group exhibited an average ΔE^00^ value within the clinically acceptable range. These findings suggest that 3D-printed restorations have superior color stability compared to the other groups.

### 3.3. Evaluation of the Color Change Differences (ΔE^00^) and Fracture Resistance (K_IC_)

In this study, the relationship between the color change differences (ΔE^00^) measured before and after thermal cycling and fracture resistance (K_IC_) was analyzed. A weak negative correlation was found across all groups (r = −0.208); however, this relationship was not statistically significant (*p* = 0.269). The weakest negative correlation was observed in the 3D group (r = −0.042), while the FC group showed a weak negative correlation (r = −0.321), and the IC group exhibited a moderate negative correlation (r = −0.418). These findings indicate that there is no strong or statistically significant relationship between color change and fracture resistance.

### 3.4. Finite Element Stress Analysis (FEA) Findings

In this study, FEA analysis was performed on a sample model from each experimental group to assess the accuracy and consistency of the measured fracture resistance (K_IC_) values in a simulation environment that mimics intraoral conditions. In this analysis, von Mises stress values (MPa) and displacement amounts (mm) were evaluated.

**Figure 3:**
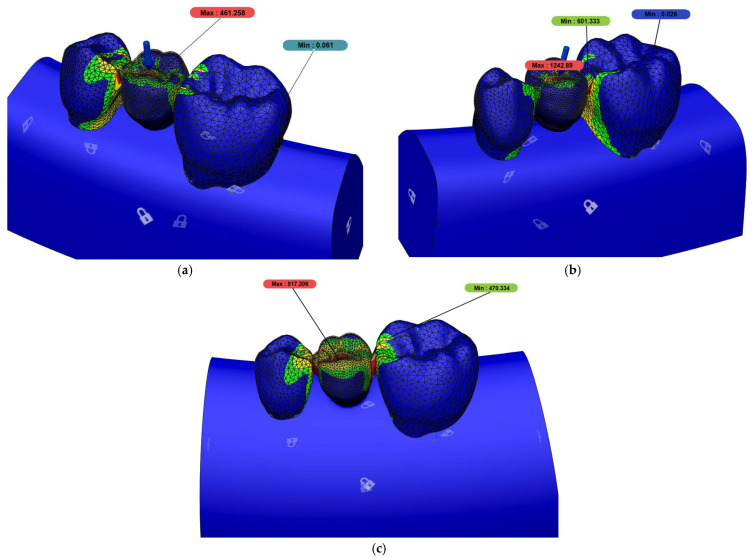
(**a**) Maximum von Mises stress value for 3D group, (**b**) maximum von Mises stress value for FC group, and (**c**) maximum von Mises stress value for IC group.

According to the static force analysis results, the highest von Mises stress value was observed in the woven fiber and direct composite (FC) group, while the lowest stress value was recorded in the 3D-printed restorations (3D) group. The indirect composite (IC) group exhibited a moderate level of stress. The differences among the groups were found to be statistically significant (Table 4) (Figure 3).

**Figure 4:**
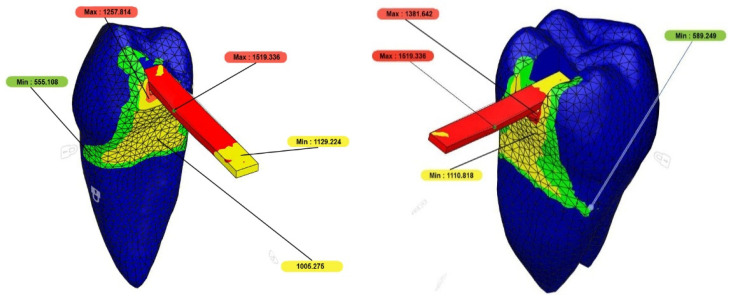
In the FC group, the force is concentrated on the woven fiber.

In all groups, stress accumulation was predominantly observed on the pontic second premolar, whereas in the FC group, stress was particularly concentrated on the woven fiber (Figure 4). When evaluating the stress distribution in adjacent teeth, it was observed that less stress accumulated in the first premolar, while higher stress levels were recorded in the first molar.

**Figure 5:**
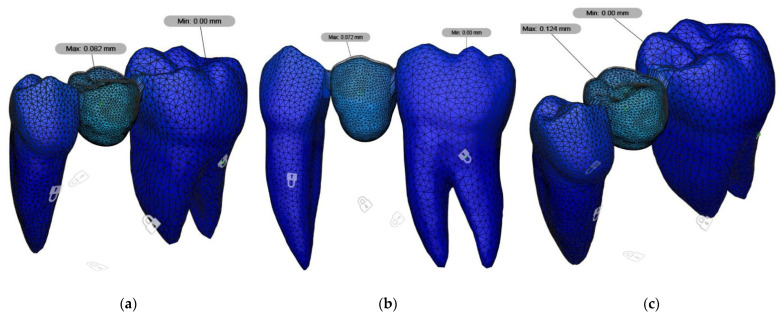
(**a**) Displacement for 3D group, (**b**) displacement for FC group, and (**c**) displacement for IC group.

Displacement analysis showed the highest apical displacement in the 3D group, while the IC group exhibited a higher degree of displacement. The FC group demonstrated the lowest displacement, with minimal movement detected within the high-molecular-weight glass woven fiber embedded in the resin composite. However, the differences in displacement among the groups were not statistically significant (Table 5) (Figure 5).

When assessing the correlation between the fracture resistance (K_IC_) values obtained from mechanical tests and von Mises stress results in the simulation environment, a strong and statistically significant positive correlation was found. These findings indicate that the mechanical test results are consistent with the loading conditions in the simulation environment.

## 4. Discussion

Single-tooth deficiencies are one of the most common developmental anomalies encountered today and can occur due to factors such as congenital causes, trauma, or tooth extraction. Among the minimally invasive restoration options for the treatment of single-tooth deficiencies, the most used methods today are direct composite restorations reinforced with fiber mesh and indirect resin composite restorations [1,13,14]. Studies in the literature have investigated the necessity of preparation in adjacent teeth and its long-term prognosis in posterior single-tooth restorations using either fiber-reinforced or CAD/CAM-fabricated restorations. Van Heumen et al. (2010) evaluated the five-year clinical success of adhesive bridges in the posterior region, and their findings suggested that adhesive bridges could be a successful option in the posterior region, provided that proper cavity preparation was performed in the abutment teeth and a sufficient volume of composite material was used [15]. Therefore, in this study, different treatment alternatives for the restoration of a missing mandibular second premolar were evaluated.

However, surface-retained designs showed higher incidences of retention loss and delamination [15]. The variation in success rates between studies may be influenced by differences in abutment preparation designs and cavity geometry. Inlay-retained designs, as utilized in this study, allow for better load distribution compared to surface-retained designs, thereby improving mechanical stability and longevity. Similarly, another study which aimed to investigate the effect of different cavity designs on the load-bearing capacity of adhesive bridges found that surface-retained designs exhibited lower durability [16]. Based on these findings, inlay-retained cavity designs were utilized for the abutment teeth in this study.

There are also studies in the literature that examine the effect of different fiber placements on the mechanical strength of adhesive bridges. In a study conducted by Tacir et al., it was reported that the highest fracture resistance was 1085.87 N in the group with an inlay-supported design and a single-layer fiber mesh, regardless of fiber quantity and orientation [16]. In this study, the mean fracture resistance for the FO group was measured as 1057.92 N, while the value determined through finite element stress analysis was 1242.65 MPa. These findings are consistent with similar studies in the literature, supporting that inlay-supported designs can be an effective option for enhancing the mechanical strength of adhesive bridges without the need to increase the number of fiber layers. The possible reasons of these minor differences in the fracture resistance values may be attributed to several factors, including variations in the fiber mesh types, fiber volume content, resin matrix composition, and the mechanical properties of the materials used. Furthermore, differences in connector dimensions and pontic design between studies could influence the load distribution patterns and ultimately affect the mechanical performance of adhesive bridges. Furthermore, complete coverage of fiber mesh with composite is a critical requirement to ensure mechanical stability, biocompatibility, and long-term success of the restoration [17]. Additionally, to maintain occlusal stability and ensure compatibility with antagonist teeth, the composite material must be applied at a specific thickness. It has been suggested that using two layers of fiber placement may reduce the available volume for composite, negatively affecting material distribution and polymerization efficiency. This, in turn, could weaken the mechanical durability of the restoration and lead to marginal misalignment issues. Therefore, in this study, a single-layer fiber placement was preferred to allow adequate space for composite material and to preserve the ideal mechanical and aesthetic performance of the restoration. Similar to this study, Jadhav et al. has shown that a single layer of fiber woven placement exhibited higher fracture toughness when compared with none or two layers, which is consisted with our findings [18].

In a study conducted by Ellakwa et al., the flexural strength of fiber-reinforced composites was evaluated, and it was determined that glass fibers provided higher flexural strength compared to polyethylene fibers [19]. These findings indicate that glass fibers are more effective than polyethylene fibers in enhancing the mechanical properties of composite materials. Another in vitro study by Candan et al. reported that nanofill composites reinforced with glass fibers (78% wt) exhibited increased flexural strength. Their evaluation showed that fractures were limited to stress areas where fibers were present and that glass fibers functioned as a fracture-arresting barrier [20]. For these reasons, in this study, Interlig fiber mesh (Angelus Odontologia, Londrina, Brasil) was chosen due to its high glass fiber content (60% glass fiber, 40% resin matrix), which provides resistance to vertically applied forces and biomechanical support to dental structures. Additionally, its pre-impregnated structure enhances adhesion compatibility with resin composites while improving shear and tensile strength due to a reduced elastic modulus. This structure also facilitates ease of application and improves adaptation to cavity walls. Therefore, the findings proved the fact that a high glass fiber/resin matrix ratio exhibited better fracture toughness (1242 Mpa) when compared to 3D resin (Varseo Smile Crown Plus—25% wt) with a lower filler/resin matrix ratio.

Although various indirect composite systems are available today, resin composite systems specifically designed for the fabrication of indirect restorations remain limited. For this reason, in this study, Signum (Heraus Kulzer, Hanau, Germany), an indirect composite material exclusively intended for indirect restorations, was selected. A long-term clinical study evaluating the success of this material followed 113 inlay restorations placed in 30 patients over a 12-year period [21]. The study reported a 12% failure rate; however, despite changes in surface and marginal properties, the restorations demonstrated functionally satisfactory clinical outcomes. Additionally, an in vitro study demonstrated that Signum composite had an average fracture resistance of 608.25 N, a finding consistent with the fracture resistance values obtained in the present study [22].

In this study, the samples were subjected to a total of 5000 thermal cycles between 5 and 55 °C, simulating six months of aging. At the end of this process, color measurements revealed that the lowest ΔE^00^ change was observed in the 3D restoration group. Despite the application of a common glazing procedure across all groups, the superior color stability in the 3D group may be attributed to the completion of polymerization at an optimal level and the absence of residual monomers. Moreover, differences in thermal aging protocols used across studies, such as the number of cycles, dwell times, and temperature ranges, can significantly impact the degree of color change observed. In addition, variations in the composition and filler content of the restorative materials, as well as differences in the glaze or surface sealant properties, even the color of the selected resin, may affect water absorption and hydrolytic degradation, leading to discrepancies in color stability outcomes [23,24].

Finite element stress analysis (FEA) results indicated that while the stress distribution patterns in the 3D and IC models were nearly identical, stress was predominantly concentrated on the fiber mesh in the FC group. One of the primary purposes of using fiber mesh is to absorb occlusal forces due to its high elastic modulus and to distribute these forces by flexing in the inciso-gingival direction, thereby transferring them to the periodontal structures for dissipation. A similar study demonstrated through FEA that, in the restoration of mandibular premolars, forces were absorbed by the fiber mesh rather than being transmitted along the natural tooth axis when fiber reinforcement was used alongside composite resin. Furthermore, groups utilizing fiber mesh exhibited superior fracture resistance and von Mises stress distribution compared to composite resin-only restorations [25].

Von Mises stress distribution analysis revealed that stress loads were concentrated on the pontic second premolar in all groups. In the FC group, the force was particularly concentrated on the fiber mesh, reaching 1519.336 MPa. This finding suggests that fiber-reinforced adhesive restorations enhance load-bearing capacity and contribute to a more even distribution of forces throughout the restoration. A similar study in the literature reported high stress accumulation at the junctions of bridge components, with the highest forces concentrated between the second premolar and first molar [26]. This indicates that the connection area serves as a potential fracture point, emphasizing the importance of restoration design in addressing these critical regions.

The literature indicates that the maximum stress values for posterior teeth range between 500 and 900 N [27]. Considering this threshold, the findings of this study suggest that the mechanical properties of the 3D restorations need further improvement for use in the posterior region. Incorporating fiber structures into 3D-printed dental restorations could be a promising approach to enhance their mechanical performance. Although the addition of fibers may not significantly affect the degree of polymerization, it is likely to substantially improve the interlayer bonding strength, which is a known limitation in additively manufactured materials. Strengthening the adhesion between printed layers could reduce the risk of delamination and increase fracture resistance. Future studies investigating fiber-reinforced 3D printing techniques, such as co-printing fibers with resins or embedding fibers during the printing process, could offer valuable insights for optimizing the mechanical properties of 3D-printed restorations. In contrast, the FC and IC groups demonstrated sufficient mechanical durability, making them suitable for clinical application in posterior load-bearing areas.

The differences in fracture resistance and color stability among the FC, IC, and 3D groups can be attributed to the intrinsic properties of the materials and fabrication methods. The woven fiber and direct composite group (FC) showed the highest fracture resistance due to the reinforcing effect of the fiber mesh, which effectively redistributed occlusal forces. The indirect composite group (IC) demonstrated moderate fracture resistance, benefitting from high filler content and controlled laboratory polymerization. The 3D-printed restoration group (3D) exhibited the lowest fracture resistance but superior color stability, likely due to optimal polymerization during post-curing and the absence of residual monomers that can induce discoloration over time.

## 5. Conclusions

One of the initial hypotheses of this study was that adhesive restorations fabricated using three different methods would exhibit similar fracture resistance in compression tests, with no statistically significant differences between them. Based on the study findings, this hypothesis was rejected.

The second hypothesis proposed that the color changes (ΔE^00^) of adhesive restorations after the aging process would remain below the clinically perceptible threshold, with no significant differences among the groups. However, the results showed that the FC group had the highest ΔE^00^ values, displaying significant differences compared to both the 3D and IC groups. The 3D and IC groups, on the other hand, were similar, with no significant differences between them. Only the 3D group exhibited average ΔE^00^ values within the clinically acceptable range. Thus, this hypothesis was also rejected.

The third hypothesis suggested that the static and dynamic compression strength values calculated using finite element stress analysis (FEA) would be consistent with the experimental results. This hypothesis was accepted.

Based on these findings, the following recommendations should be considered for future studies:

- Fiber-reinforced restorations were found to enhance mechanical durability. The use of fiber-reinforced systems may be particularly beneficial in the posterior region, where masticatory forces are intense.
- The greatest color changes after aging were observed in fiber-reinforced restorations. Therefore, the development of new monomer structures and polymerization processes that improve color stability in these restorations is necessary.
- Additionally, the mechanical properties of restorations produced using 3D dental printing technology need further enhancement for use in posterior regions. However, these materials show promise for single-tooth replacement in the anterior region, where occlusal forces are lower. Further research is needed to evaluate their bonding strength, wear resistance, and long-term clinical performance.

## Figures and Tables

**Table 1:** Mechanical properties of the dental materials and soft and hard tissues used in the FEA.

Material	Young’s Modulus (GPa)	Poisson’s Ratio	Shear Modulus (MPa)	Density (g/cm^3^)
Enamel [7]	84.094	0.33	40,000.000	2.600
Dentin [7]	18.600	0.31	30,800.000	2.120
Pulp [8]	0.0028	0.45	1700.000	1.188
Cementum [7]	4.402	0.31	23,499.999	1.240
Periodontal ligament [9]	0.0689	0.45	2.100	1.8
Oral mucosa [10]	0.083	0.40	0.389	1.097
Cortical bone [11]	14.500	0.45	51.600	1.392
Resin cement [12]	12.890	0.31	4616.000	1.380
Woven fiber *	X. Y: 7000 Z: 46,000	X. Y: 0.29 Z: 0.30	131 (±15)	1.80
Resin composite *	20.740	0.35	1700.00	1.188
3D printer resin *	31.823	0.35	2000.00	1.395

* Product information is taken from the manufacturer’s safety data sheet.

**Table 2:** Averages of fracture strength values of groups.

Groups	N	Mean (N)	*p* Value
3D	10	396.34	0.001
FC	10	1057.91	
IC	10	763.11	
Enamel		1244.01	
Dentin	N/A *	449.05	
Cortical bone		170.50	

* N/A: Enamel, dentin, and bone values were added to compare the results with natural tissues in terms of compatibility and proximity to hard dental tissue values.

**Table 3:** Averages of color change values of groups.

Groups	N	Mean (ΔE^00^)	*p* Value
3D	10	1.7059	0.129
FC	10	3.6459	
IC	10	2.87187	

**Table 4:** Maximum von Mises stress values of the groups.

Groups	N	Von Mises Stress Value (MPa)	*p* Value
3D	10	461.25	<0.05
FC	10	1242.64	
IC	10	917.24	
Enamel		150.00	
Dentin	N/A *	138.87	
Cortical bone		19.00	

* N/A: Enamel, dentin, and bone values were added to compare the results with natural tissues in terms of compatibility and proximity to hard dental tissue values.

**Table 5:** The amount of displacement of the groups.

Groups	N	Displacement (mm)	*p* Value
3D	10	0.082	>0.05
FC	10	0.072	
IC	10	0.124	

## Data Availability

Data are contained within the article.
